# Rational design of efficient electrode–electrolyte interfaces for solid-state energy storage using ion soft landing

**DOI:** 10.1038/ncomms11399

**Published:** 2016-04-21

**Authors:** Venkateshkumar Prabhakaran, B. Layla Mehdi, Jeffrey J. Ditto, Mark H. Engelhard, Bingbing Wang, K. Don D. Gunaratne, David C. Johnson, Nigel D. Browning, Grant E. Johnson, Julia Laskin

**Affiliations:** 1 Physical Sciences Division, Pacific Northwest National Laboratory, PO Box 999, MSIN K8-88, Richland, Washington 99352, USA; 2 Department of Chemistry, University of Oregon, Eugene, Oregon 97403, USA; 3 Environmental Molecular Sciences Laboratory, Pacific Northwest National Laboratory, Richland, Washington 99352, USA

## Abstract

The rational design of improved electrode–electrolyte interfaces (EEI) for energy storage is critically dependent on a molecular-level understanding of ionic interactions and nanoscale phenomena. The presence of non-redox active species at EEI has been shown to strongly influence Faradaic efficiency and long-term operational stability during energy storage processes. Herein, we achieve substantially higher performance and long-term stability of EEI prepared with highly dispersed discrete redox-active cluster anions (50 ng of pure ∼0.75 nm size molybdenum polyoxometalate (POM) anions on 25 μg (∼0.2 wt%) carbon nanotube (CNT) electrodes) by complete elimination of strongly coordinating non-redox species through ion soft landing (SL). Electron microscopy provides atomically resolved images of a uniform distribution of individual POM species soft landed directly on complex technologically relevant CNT electrodes. In this context, SL is established as a versatile approach for the controlled design of novel surfaces for both fundamental and applied research in energy storage.

## Abstract (web summary)

The design and understanding of electrode–electrolyte interfaces is important for the development of improved energy storage devices. Here, the authors study the controlled deposition of molybdenum polyoxometalate anions onto carbon nanotube electrodes, and show this can result in increased specific capacitance.

## Graphical abstract

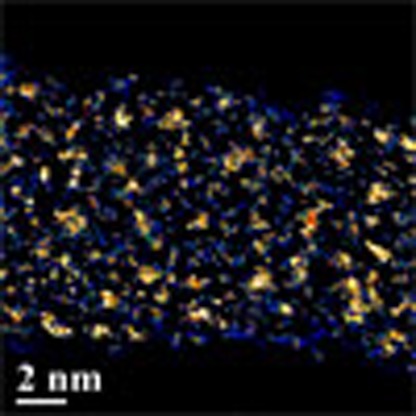

The development of improved materials for efficient energy storage is at the forefront of both fundamental and applied research in energy technology. Substantial progress both in understanding underlying phenomena and device fabrication is required to increase the charge-discharge capacity, rate and life cycle of energy storage devices while decreasing the cost associated with their manufacture^1^,^2^. Hence, the race to develop high performance, ecofriendly, low-cost energy storage devices is progressing along various parallel tracks. One familiar track is the development and optimization of the hybrid battery which is usually comprised of lithium ion batteries (Li-ion) coupled with supercapacitors^1^,^3^,^4^,^5^. The main challenge with Li-ion batteries is to provide improved power density and fast charge rates at a reduced price per kW h^−1^, whereas supercapacitors need improvement in their energy densities so that the latter can respond to the coupled battery during combined operation^5^,^6^,^7^,^8^,^9^. In the past decade, both the power and energy density of supercapacitors have been significantly improved by combining non-Faradaic electrochemical double-layer capacitance and Faradaic pseudocapacitance^10^,^11^ through a combination of high surface area carbon and metal oxides integrated at a molecular level^12^,^13^. Redox-supercapacitor electrodes have been fabricated by the direct transfer of redox pseudocapacitive materials from the solution phase onto electrodes using direct painting^14^, ambient air spray^15^, chemical vapour deposition^16^, atomic layer deposition^17^ and electrodeposition^18^. Electrodes prepared via such methods typically contain both electrochemically active components and inactive counter ions at the electrode–electrolyte interface (EEI). Polyoxometalates (POMs) are attractive redox species because of their stability, multi-electron redox activity^19^,^20^ and strong interactions with electrodes^21^,^22^,^23^. Here, we demonstrate that fabrication of an energy storage device using uniformly distributed discrete POM anions without aggregate formation is an important breakthrough towards achieving superior performance not possible with conventional preparation techniques.

We employ soft landing (SL) of mass-selected ions for the preparation of well-defined electrode surfaces with an unprecedented level of control. SL enables the uniform deposition of ions with specific composition, charge state and kinetic energy^24^,^25^,^26^,^27^,^28^,^29^ which is critical to gaining fundamental understanding of interfacial phenomena relevant to catalysis and materials science^27^,^30^,^31^,^32^,^33^. Previously, SL and reactive landing have been used to functionalize carbon nanotubes (CNTs), graphene, Si and Au surfaces to study electrochemical properties of well-defined deposited ions^34^,^35^,^36^,^37^,^38^. Notably, SL allows us to fabricate electrode surfaces for energy storage devices that are difficult to prepare using conventional solution- and vacuum-based deposition techniques. This provides a unique opportunity to design efficient and stable EEI by (i) studying the inherent activity and efficacy of precisely defined electro-active species; (ii) understanding the effect of electrochemically inactive counter-ions at the EEI on overall device performance; and (iii) identifying pathways to enhance the overall efficacy and selectivity of reactions of interest.

We demonstrate that SL of only small amounts of redox-active species results in dramatically enhanced total specific capacitance and superior long-term stability of carbon-based electrodes. We also show that aggregation is largely responsible for the reduced performance of POM-based supercapacitors fabricated from solution.

## Results

### Role of ion soft landing in electrode fabrication

**Figure 1:**
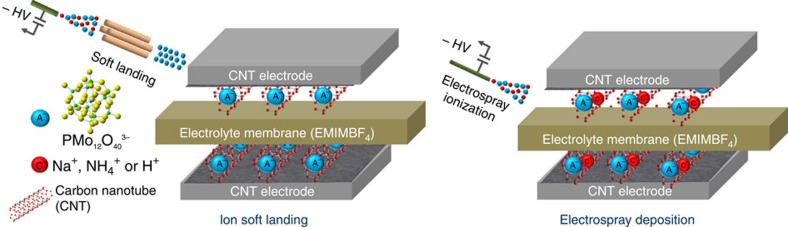
Electrode–electrolyte interfaces of fabricated supercapacitor devices. Schematic representation of EEI of redox-supercapacitors fabricated using SL and ambient ESD. Note the absence of countercations in the device prepared by SL.

Using a specially designed high-flux ion SL instrument (see Supplementary Fig. 1)^39^, we fabricated macroscopic energy storage devices and investigated the role of anion–cation interactions and aggregate formation on the performance of POM-based supercapacitors. The SL instrument is equipped with a dual ion funnel interface, which increases the deposition rate of ions (refer to Methods section for a detailed description of our SL instrument). Specifically, redox-supercapacitor devices were fabricated by immobilizing mass-selected PMo_12_O_40_^3−^ onto CNT-coated carbon electrodes (CNT electrodes) and compared with a non-Faradaic CNT-based device. SL can be performed either in vacuum or at ambient conditions on a benchtop^29^. In this study, vacuum-based SL was used to achieve selective deposition of PMo_12_O_40_^3−^ anions while excluding other charge states of POM (2- and 1-) produced by electrospray ionization (ESI) (see Supplementary Fig. 2). Predetermined amounts of POM were deposited onto CNT electrodes either using SL or ambient electrospray deposition (ESD) of Na_3_[PMo_12_O_40_], (NH_4_)_3_[PMo_12_O_40_] or H_3_[PMo_12_O_40_] solutions (Fig. 1). In ESD, micron size charged droplets of solvent containing POM anions and counter-cations, are deposited onto electrodes with a set deposition rate controlled by the flow of POM solution. Therefore, the ions deposited using ESD are neither mass-selected nor charge-selected. Ambient ESD along with its analogue, ambient air spray, that does not use high voltage are commonly used fabrication methods for thin film deposition on surfaces and electrodes^40^,^41^,^42^,^43^,^44^,^45^. It has been demonstrated that deposition of small microdroplets by ESD produces more uniform films in comparison with bulk solution drop casting making it a preferred deposition method. Furthermore, the ability to control the amount of deposited material by ESD enables a quantitative comparison with SL.

In addition, we fabricated supercapacitors by drop casting Na_3_[PMo_12_O_40_] solution (DRP-NaPOM-CNT) at different POM loadings and compared the total specific capacitance of these electrodes with ones prepared by ESD. Consistent with the literature^46^,^47^,^48^, the results demonstrated the superior performance of ESD (see Supplementary Fig. 3) making it a suitable benchmarking technique for comparison with SL.

A total of five different electrode configurations were employed in this study: (i) pristine CNT electrode (pCNT); (ii) SL electrode containing a known amount of charge- and mass-selected PMo_12_O_40_^3−^ (SL-CNT); (iii) ESD electrode with Na_3_[PMo_12_O_40_] (ESD-NaPOM-CNT); (iv) ESD electrode with (NH_4_)_3_[PMo_12_O_40_] (ESD-NH_4_POM-CNT); and (v) ESD electrode with H_3_[PMo_12_O_40_] (ESD-HPOM-CNT).

This study demonstrates that SL-CNT containing only a small amount of PMo_12_O_40_^3−^ (∼50 ng POM on ∼25 μg of the CNT material) have remarkably higher total specific capacitance and superior long-term stability compared with ESD electrodes. High-angle annular dark-field scanning transmission electron microscopy (HAADF-STEM) demonstrates that SL electrodes exhibit an extremely uniform and narrow distribution of discrete 0.75 nm diameter [PMo_12_O_40_] clusters without any agglomeration. In contrast, the formation of aggregates and agglomerated POM in the presence of counter cations and solvent is observed on ESD electrodes. On the basis of these observations, we propose that aggregation is largely responsible for the reduced performance of POM-based supercapacitors fabricated from solution. Our results demonstrate that higher performance and longer stability can be achieved in redox-supercapacitors by uniform deposition of discrete redox-active species without strongly coordinating yet inactive counter ions or solvent at the EEI.

### Electrochemical performance of fabricated devices

The supercapacitors were fabricated as described in the methods section using a 1-ethyl-3-methylimidazolium tetra fluoroborate (EMIMBF_4_) ionic liquid membrane as a separator. The effect of the POM deposition technique and loading on total specific capacitance and stability of the fabricated devices was assessed. Our previous study showed that soft-landed PMo_12_O_40_^3−^ remain intact and redox active on surfaces^49^. The redox activity of PMo_12_O_40_^3−^ in the EMIMBF_4_ ionic liquid was confirmed by examining the cyclic voltammograms (CVs) of different POM salts on glassy carbon (GC) electrodes (see Supplementary Fig. 4). A rectangular CV over different potential ranges was observed for supercapacitors fabricated using pCNT (see Supplementary Fig. 5) confirming the stability of the electrolyte membrane and the fabricated supercapacitor over a technologically relevant potential range^50^,^51^,^52^.

**Figure 2:**
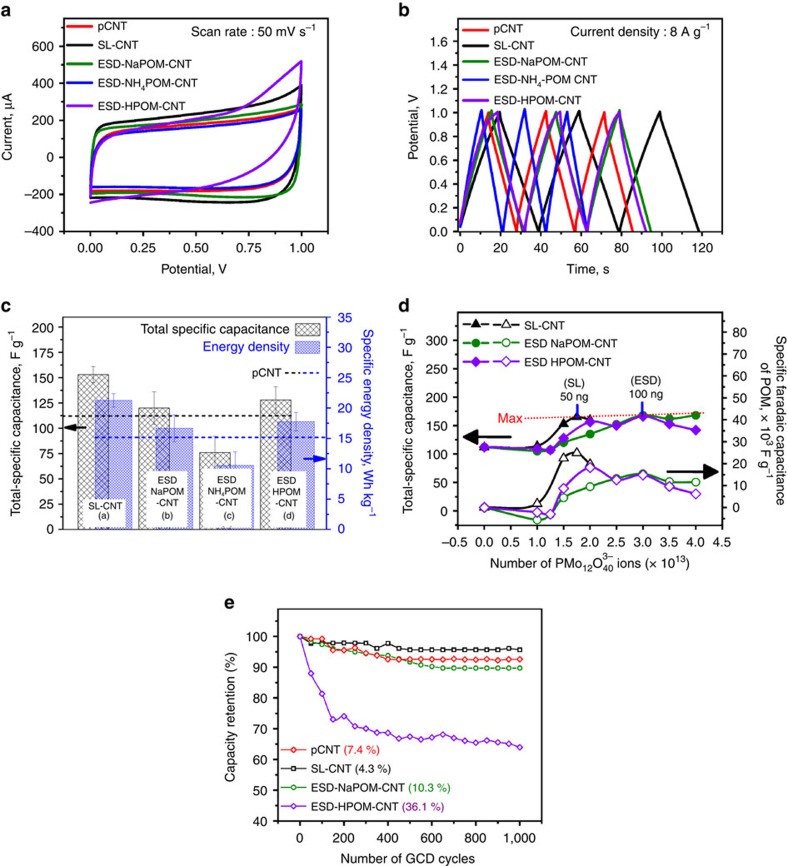
Superior performance of SL supercapacitors. Characterization of supercapacitors fabricated with pCNT, SL-CNT, ESD-NaPOM-CNT, ESD-NH_4_POM-CNT and ESD-HPOM-CNT and an EMIMBF_4_ based membrane as a separator: (**a**) representative CV and (**b**) galvanostatic charge-discharge (GCD) curves. (**c**) Comparison of the total specific capacitance and specific energy density. Approximately 1.5 × 10^13^ PMo_12_O_40_^3−^ were deposited in each case. (**d**) Effect of PMo_12_O_40_^3−^ loading on the total specific capacitance (black) and specific Faradaic capacitance (green). (**e**) Capacity retention as a function of the number of GCD cycles. Current density: 8 A g^−1^.

The CVs of supercapacitors fabricated with pCNT, SL-CNT, ESD-NaPOM-CNT and ESD-NH_4_POM-CNT (Fig. 2a) are rectangular in shape indicating that they exhibit an ideal capacitive-like behaviour. The respective galvanostatic charge-discharge (GCD) curves (Fig. 2b) are almost symmetrical triangles without any significant voltage drop related to internal resistance during the changing of polarity, suggesting fast transmission of ions at the EEI. However, ESD-HPOM-CNT showed neither rectangular CV (Fig. 2a) nor triangular GCD curves (Fig. 2b) which may be attributed to the presence of additional Faradaic reactions. The increase in current response closer to 1 V in the CV of ESD-HPOM-CNT indicates the presence of a side reaction such as oxygen evolution^53^.

The GCD measurements were performed in triplicates and a total specific capacitance of 112±12, 153±8, 120±16, 76±16 and 128±13 F g^−1^ and specific energy densities of 15.0±1.7, 21.3±1.1, 16.7±2.2, 10.6±2.2 and 17.75±1.5 Wh kg^−1^ were obtained for the pCNT, SL-CNT, ESD-NaPOM-CNT, ESD-NH_4_POM-CNT and ESD-HPOM-CNT, respectively, using equations 1 and 2. Similar values were also calculated from CV data using equation 3 indicating that the supercapacitors perform well both in constant potential (CV) and constant current mode (GCD). Of note, the total specific capacitance and energy density of pCNT are in agreement with literature values^54^, which confirms the reliability of the baseline pCNT and the device fabrication method adopted in this study. The lack of dependence of the specific capacitance on the scan rates (see Supplementary Figs 6–9 and 11) indicates that, except for ESD-HPOM-CNT, the mobility of the ions and surface pseudocapacitance of unmodified and modified electrodes remain constant and stable over a wide range of charge-discharge rates of interest to energy storage applications. The ESD-HPOM-CNT showed an unstable capacitance over different scan rates (see Supplementary Figs 10 and 11), which may be attributed to the presence of additional Faradaic capacitance as discussed earlier.

The total specific capacitance and energy density of the SL-CNT (Fig. 2c) is ∼36, 27, 101 and 20% higher than the values obtained for pCNT, ESD-NaPOM-CNT, ESD-NH_4_POM-CNT and ESD-HPOM-CNT, respectively. Therefore, the efficient participation of SL-PMo_12_O_40_^3−^ during redox reaction in the absence of counter cations contributes to the enhanced capacitance of SL-CNT. The 30% decrease and the 7 and 14% increase in the total specific capacitance of ESD-NH_4_POM-CNT, ESD-NaPOM-CNT and ESD-HPOM-CNT, respectively, in comparison with pCNT clearly indicate the important role of counter cations that are absent in SL-CNT. For example, NH_4_^+^ is known to strongly adsorb on carbon^55^,^56^, which may decrease the electrochemically active surface area and reduce the non-Faradaic capacitance of the CNT surface along with the Faradaic capacitance of POM. Similar specific power densities of ∼4 kW kg^−1^ were obtained for both pristine and modified CNT electrodes using equation 4. However, the SL-CNT with a similar specific power density is characterized by a higher specific energy density demonstrating the improved performance of this electrode.

### Effect of POM loading on specific capacitance

GCD measurements were also carried out with the SL-CNT, ESD-NaPOM-CNT and ESD-HPOM-CNT containing different loadings of POM (Fig. 2d). The ESD-NH_4_POM-CNT was excluded in this further study as it showed lower capacitance than the pCNT. The SL-CNT achieved its maximum total specific capacitance of ∼160 F g^−1^ with 1.75 × 10^13^ POM ions. Meanwhile, the ESD-NaPOM-CNT required almost twice the amount of POM (3 × 10^13^ ions) and the ESD-HPOM-CNT required 2 × 10^13^ ions compared with the SL-CNT to achieve a similar maximum total specific capacitance. This demonstrates the higher efficacy of SL-CNT. For both SL and ESD electrodes, further addition of POM above the required maximum amount resulted in a decrease in the total specific capacitance.

It is remarkable that the maximum total capacitance was obtained only with 1.75 × 10^13^ SL-PMo_12_O_40_^3−^ (∼50 ng POM on 25 μg of the active CNT material) demonstrating the unexpectedly high contribution of nanogram quantities of pure PMo_12_O_40_^3−^ to the total capacitance. The Faradaic component of the total specific capacitance (Fig. 2d) was calculated using equation 5. It is observed that the specific Faradaic capacitance of SL-CNT is greater than that of ESD-NaPOM-CNT and ESD-HPOM-CNT at all POM loadings. The observed decrease in capacitance at higher coverage may be attributed to formation of additional POM layers in which underlying layers do not participate in redox activity.

The capacity retention (stability) of the pCNT, SL-CNT, ESD-NaPOM-CNT and ESD-HPOM-CNT was evaluated by performing 1,000 GCD cycles. After cycling (Fig. 2e), the capacitance of ESD-NaPOM-CNT decreased by 10.3 %, whereas only a 4.3% decrease was observed with SL-CNT indicating that SL-CNT have almost twice the lifetime of the ESD-NaPOM-CNT. The capacitance of pCNT was decreased by 7.4 % after cycling, which further confirmed the higher stability of SL-CNT. Interestingly, the ESD-HPOM-CNT showed ∼36% decrease in capacitance after 1,000 GCD cycles. The lower stability of ESD-HPOM-CNT was attributed to degradation of EEI due to the presence of side reactions as evidenced in the CVs (See Fig. 2a and Supplementary Fig. 10) or chemical degradation of EMIMBF_4_ in presence of free H^+^ ions^57^. However, the exact mechanism responsible for the lower stability of ESD-HPOM-CNT is not clear. Further characterization of the instability of ESD-HPOM-CNT is beyond the scope of this work. The poor capacity retention of ESD-HPOM-CNT made them unsuitable to compare with SL-CNT.

### Physical and chemical properties of fabricated electrodes

The exceptional performance and superior long-term stability of SL-CNT may reflect the absence of interactions between PMo_12_O_40_^3−^ ions and their strongly coordinating counterions, which facilitates the uniform distribution of discrete PMo_12_O_40_^3−^ on SL-CNT without agglomeration, or differences in the oxidation state of POM. To evaluate these possibilities, the distribution and chemical state of POM on CNT were analysed using HAADF-STEM, scanning electron microscopy (SEM) and X-ray photoelectron spectroscopy (XPS).

**Figure 3:**
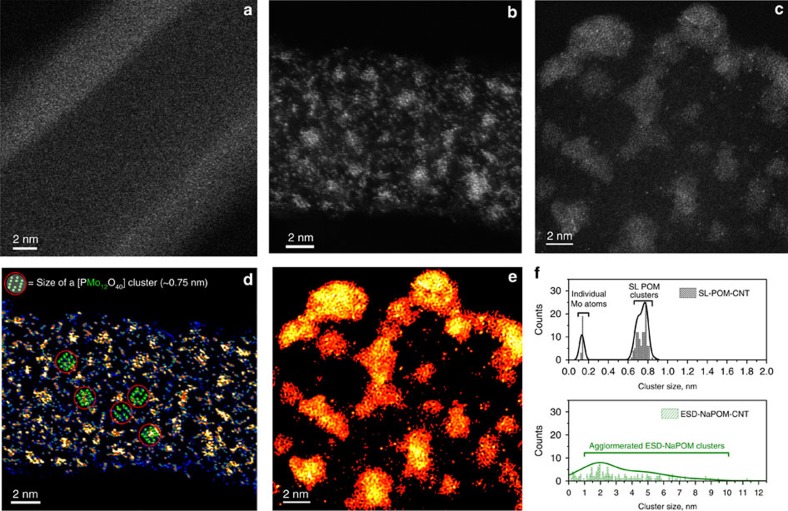
Atomically resolved STEM images of monodisperse SL POM. (**a**–**c**) HAADF-STEM images of pCNT, SL-CNT and ESD-NaPOM-CNT, respectively. (**d**,**e**) The corresponding HAADF STEM images of SL-CNT and ESD-NaPOM-CNT, respectively, (**f**) Histogram of cluster size distribution in SL-CNT and ESD-NaPOM-CNT, respectively. Approximately 1.5 × 10^13^ PMo_12_O_40_ ions were loaded on the modified CNT electrodes. Selected examples of intact SL POM clusters were mapped in **d**—red circles. Note: The raw STEM images were processed using the Gatan Microscopy Suite DigitalMicrograph software to generate colour-enhanced Z-contrast images.

Considering the fact that the ESD-NaPOM-CNT showed a similar ideal GCD behaviour and redox activity of POM anions with respect to SL-CNT, it was selected as a reference to compare with SL-CNT. Therefore, HAADF-STEM was used to examine the morphology of pCNT, SL-CNT and ESD-NaPOM-CNT at the nanometre scale (See Fig. 3a–f and Supplementary Figs 12 and 13). It is remarkable that a uniform distribution of soft-landed POM was observed directly on the complex commercially relevant CNT electrodes. A uniform and narrow size distribution of POM was observed on SL-CNT (Fig. 3b,d). The diameter of individual clusters was measured to be ∼0.74±0.04 nm (see Fig. 3f and Supplementary Figs 14 and 15) which matches the theoretical size calculated for PMo_12_O_40_^3−^ (ref. 20). STEM images indicate that PMo_12_O_40_^3−^ on SL-CNT are predominantly preserved as discrete ions without any significant agglomeration and the estimated uniform cluster coverage is 2.1 × 10^5^ POM clusters per μm^2^. Fig. 3f also shows the presence of a minor fraction of individual Mo atoms that are challenging to group as part of POM clusters (see Supplementary Discussion). In contrast, STEM images of ESD electrodes (see Fig. 3c,e,f and Supplementary Fig. 13) show features in the size range of 3–10 nm with an average size of 3.66±2.11 nm, indicating agglomeration of POM during ESD. Such aggregate formation on ESD electrodes may be attributed to the agglomeration of POM anions in the presence of counter cations (Na^+^, NH_4_^+^ or H^+^) as the solvent evaporates from the surface. The presence of nanoscopic aggregates consisting of low-conductivity cation-POM assemblies at the EEI may decrease the overall interfacial surface area and impart additional contact resistance, which would decrease the overall capacitance of the supercapacitor. Thus, STEM images clearly show the agglomeration of POM in the ESD electrodes and a uniform distribution of individual discrete [PMo_12_O_40_] clusters in the SL electrodes, which explains both the lower performance of ESD-NaPOM-CNT and higher performance of SL-CNT.

**Figure 4:**
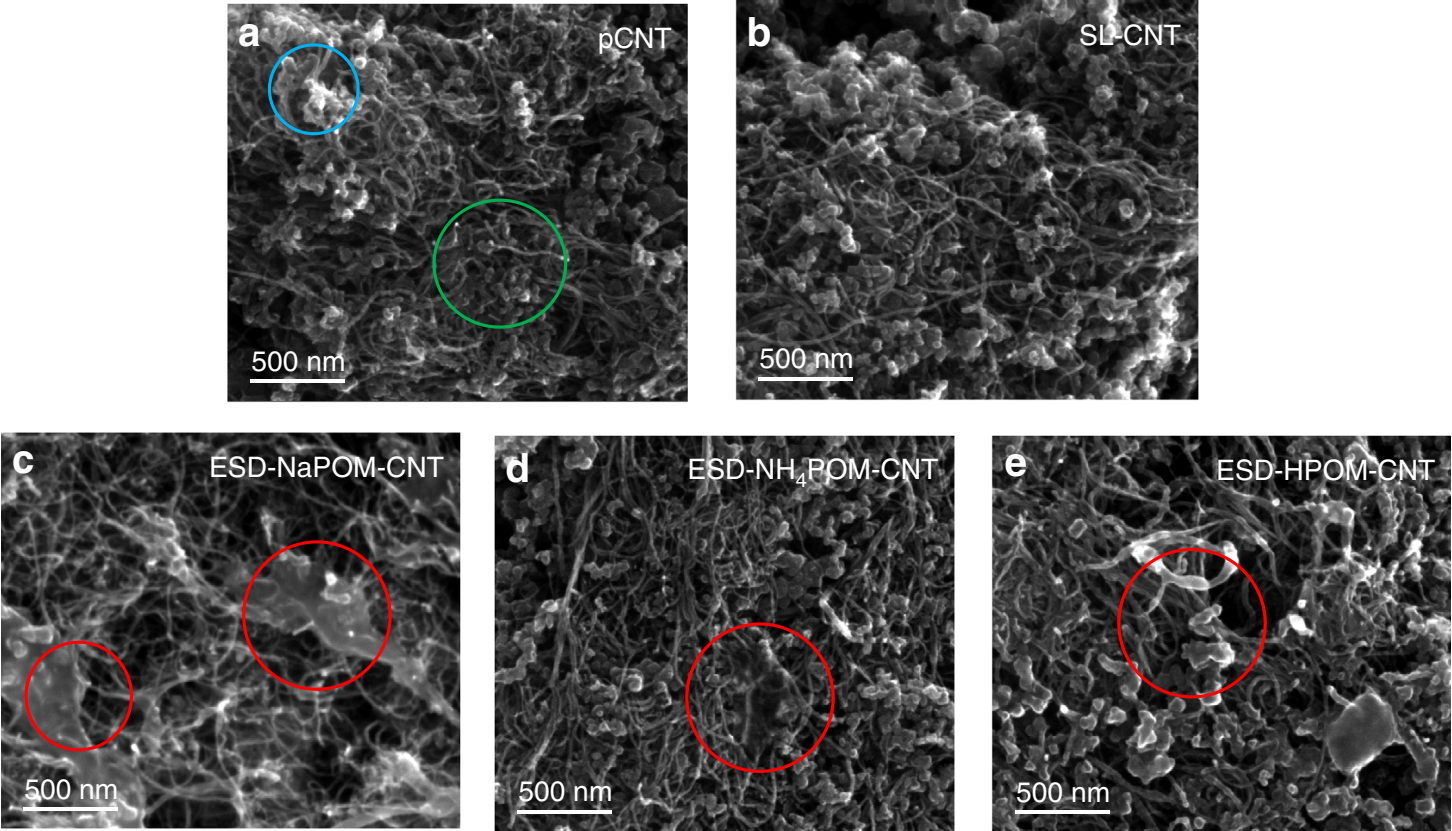
POM aggregate formation at the microscopic scale. Scanning electron micrographs of (**a**) pCNT (**b**) SL-CNT (**c**) ESD-NaPOM-CNT (**d**) ESD-NH_4_POM-CNT and (**e**) ESD-HPOM-CNT. Approximately 1.5 × 10^13^ PMo_12_O_40_ ions were loaded in the modified CNT electrodes. Green and blue circles in **a** represent the regions of CNT and carbon fibres, respectively. Red circles in **c**–**e** highlight microscopic aggregates observed on three ESD electrodes.

In addition to STEM, we also used SEM to characterize the agglomeration of POM in ESD electrodes at the microscopic scale. SEM images of pCNT, SL-CNT, ESD-NaPOM-CNT, ESD-NH_4_POM-CNT and ESD-HPOM-CNT are presented in Fig. 4. The presence of CNTs on top of the bulk carbon fibres of the base carbon electrode is clearly visible in Fig. 4a. CNT deposition helps increase the overall electrode surface area and also lowers the contact resistance at the EEI. The diameter of the pristine CNTs is found to be ∼15 nm. The SL-CNT shows similar morphology to the pristine CNT electrode and does not exhibit any characteristic presence of POM aggregates (see Fig. 4b). Given the extremely small size of individual POM clusters (diameter ∼0.75 nm), it is not possible to image them using conventional SEM. In contrast, ESD-NaPOM-CNT, ESD-NH_4_POM-CNT and ESD-HPOM-CNT show the presence of distinct aggregates on the top layer. The diameter of the CNTs present in the ESD-NH_4_POM-CNT and ESD-HPOM-CNT electrode are increased to ∼50 nm from ∼15 nm, indicating that the aggregates are preferentially formed around the outside walls of the CNTs. Formation of aggregates of POM clusters in the ESD electrodes observed with SEM analysis is consistent with the agglomeration of POM seen in the STEM analysis. Again, such aggregate formation on the electrode surface post ESD may be attributed to the agglomeration of POM in the presence of its counter cations (Na^+^, NH_4_^+^ and H^+^) as the solvent evaporates. Aggregates were not observed on the SL electrode at similar coverage. Assuming that the aggregates are composed of POM salt with lower electrical conductivity^58^,^59^, the presence of the microscopic aggregates at the EEI may significantly decrease the overall interfacial surface area and impart additional contact resistance, which would decrease the overall capacitance of the device.

**Figure 5:**
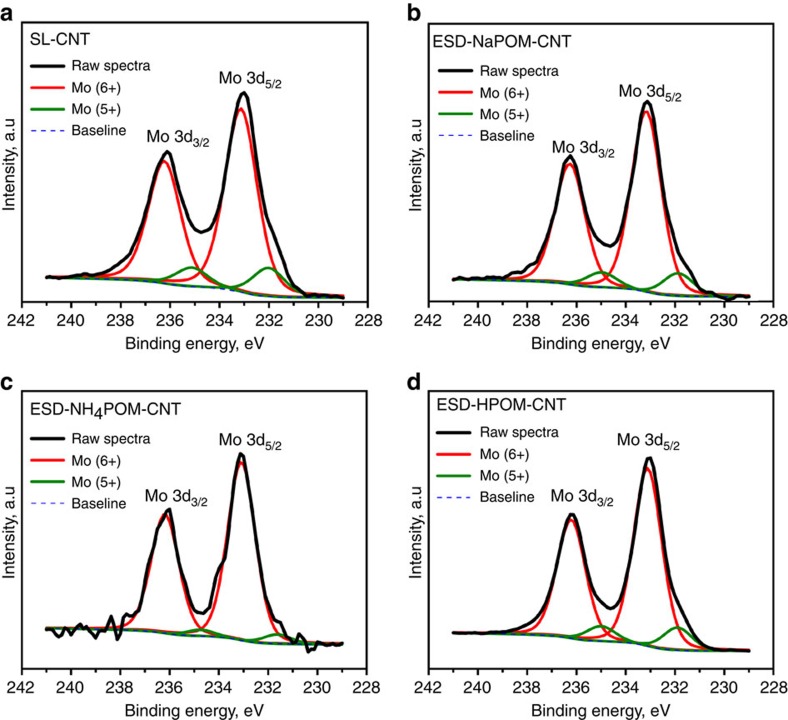
Chemical state of SL and ESD electrodes. Mo 3*d* spectra of (**a**) SL-CNT (**b**) ESD-NaPOM-CNT (**c**) ESD-NH_4_POM-CNT and (**d**) ESD-HPOM-CNT. Approximately 1.5 × 10^13^ POM ions were loaded in each case.

We also used XPS to determine the oxidation state of Mo in SL and ESD electrodes. Figure 5a–c shows Mo 3*d* XPS spectra of SL- CNT, ESD-NaPOM-CNT, ESD-NH_4_POM-CNT and ESD-HPOM-CNT. All Mo 3*d* XPS spectra exhibit the characteristic 3*d*_5/2_ and 3*d*_3/2_ doublet caused by spin–orbit coupling of the Mo 3*d* orbitals. Deconvolution of all Mo 3*d* XPS spectra with a fixed intensity area ratio of 2:3 (corresponding to d orbital^60^,^61^) reveals two peaks for each Mo 3*d*_5/2_ and Mo 3*d*_3/2_ spin–orbit coupling. The peaks located at 232.0 eV/235.1 eV and 233.1 eV/236.2 eV in Mo (3*d*_5/2_/3*d*_3/2_) spectra correspond to Mo^5+^ and Mo^6+^, respectively, which is in agreement with literature values^62^,^63^,^64^. In addition, the characteristic binding energy separation (Δ Mo 3*d*) between the Mo 3*d*_5/2_ and 3*d*_3/2_ doublet of Mo^5+^ and Mo^6+^ is ∼3.1 eV as observed previously in the literature for Mo^5+^ and Mo^6+^ ions^65^. These XPS observations show that similar oxidation states of POM clusters on CNT are present following SL and ESD. The calculated area % of different Mo chemical states post XPS analysis of all Mo 3*d* spectra are reported in Table 1. The area % of Mo^5+^ and Mo^6+^ in both Mo 3*d*_5/2_ and 3*d*_3/2_ of the SL-CNT and ESD-NaPOM-CNT are similar indicating that the charge state of Mo is not affected in both cases. However, a slightly higher abundance of Mo^6+^ seen in the ESD-NH_4_POM-CNT may be due to charge (e^−^) transfer from POM to NH_4_^+^ (NH_4_^+^ is a Lewis acid). In addition, the C 1s spectra of different CNT electrodes were also examined, but no significant changes were observed (see Supplementary Fig. 16).

The preceding binding energy and area calculations do not reveal any substantial changes in the oxidation state of Mo in SL and ESD electrodes; however, the widths of the peaks observed on ESD and SL electrodes are noticeably different. Specifically, the full-width at half-maximum (FWHM) of peaks assigned to the 5+ and 6+ oxidation states of Mo in SL-CNT, ESD-NaPOM-CNT, ESD-NH_4_POM-CNT and ESD-HPOM-CNT are 1.6, 1.4, 1.3 and 1.4 eV, respectively. The Mo 3*d*_5/2_ and 3*d*_3/2_ lines originating from SL POM ions are broader than those observed on both ESD electrodes. It is reasonable to attribute the increased broadening in SL electrodes to the presence of electronic interactions between [PMo_12_O_40_] clusters and the CNT support for the isolated SL clusters and lower crystallinity of the material prepared by SL compared with ESD^66^,^67^,^68^. In other words, the XPS analysis (see Fig. 5 and Supplementary Fig. 15) of Mo 3*d* and C 1s peaks reveals no significant change in the oxidation state of Mo and C between SL and ESD electrodes but indicates a higher crystallinity of POM on ESD electrodes which may be caused by agglomeration. This provides further evidence, in addition to the STEM and SEM images, for the surface immobilization of discrete POM anions without counter ions or solvent using SL.

## Discussion

Collectively, evidence obtained using HAADF-STEM, SEM and XPS indicates that the uniform distribution of POM, lack of agglomeration and absence of counter ions on the electrode are responsible for the exceptionally high performance of SL-CNT. Such uniform distribution of active material on the support enables efficient access to redox active species during GCD operation and improves the overall efficacy and stability of the redox-supercapacitors. In contrast, ESD results in formation of a less active, agglomerated phase that shows reduced redox activity in comparison with isolated clusters prepared by SL. Once the agglomerated phase has formed, repartitioning of individual clusters into the ionic phase in a solid state electrolyte is likely inhibited. Our results demonstrate that higher performance and longer stability can be achieved in redox-supercapacitors by uniform deposition of discrete redox active species at the EEI.

In summary, ion SL enabled the first fabrication of high-density CNT electrodes containing monodisperse POM anions and direct atomically resolved imaging of the uniform distribution of individual redox-active species on complex commercially relevant electrodes using STEM. We present evidence that agglomeration of active species due to interaction with strongly coordinating counterions is one of the key factors affecting the performance and stability of solid-state electrochemical energy storage devices such as batteries and supercapacitors. This work clearly demonstrates, for the first time, that elimination of these interactions through mass-selection and uniform deposition of small amounts of intact active species on CNT electrodes substantially improves device performance. In addition to energy storage, SL deposition can be extended to design efficient energy conversion devices where improved charge transfer across active layers^69^ is desired. In this context, SL is established as a breakthrough deposition technology that enables the rational design of efficient EEI for energy storage applications through fundamental understanding of the key limiting factors.

## Methods

### Electrode preparation

The CNT-coated carbon fibre paper was obtained from SGL carbon GmbH (Meitingen, Germany) (SIGRACET) and cut into 1 cm^2^ pieces that were directly used in this study. Hereafter, the 1 cm^2^ pieces of carbon fibre paper will be referred to as CNT electrode. The thickness of CNT electrode is ∼300 μm and the surface loading of CNT on the electrode is ∼25 μg cm^−2^. It is assumed that only the CNT present on the top layer contribute to the non-Faradaic double layer capacitance of the surface supercapacitor fabricated in this work and the minor contribution from the layer behind the electrode interface is disregarded. Therefore, the combined weight of the CNT loading (∼25 μg cm^−2^) and immobilized [PMo_12_O_40_] were used as a total weight of the active material to calculate the total specific capacitance of the supercapacitor.

*Soft landing of mass-selected PMo_12_O_40_^3−^ anions*. The PMo_12_O_40_^3−^ anions were soft-landed onto CNT electrodes using a custom-designed ion SL instrument described in detail in previous studies^39^,^49^. See Supplementary Fig. 1. The ion SL instrument is equipped with an ESI source, a dual ion funnel, an RF-only collision quadrupole, a quadrupole mass filter (Extrel CMS, Pittsburgh, PA), and three einzel lenses that focus the ion beam onto a deposition target. A 150 μM Na_3_[PMo_12_O_40_] × H_2_O solution in methanol is introduced at a flow rate of 65 μl h^−1^ to the ESI emitter. The charged droplets produced using ESI are transferred into the vacuum system using a heated stainless steel inlet and two electrodynamic ion funnels. The desolvated ions from the funnel are then collimated by colliding with the background gas in an RF-only quadrupole (collisional quadrupole). Subsequently, the ions are mass-filtered to allow through only PMo_12_O_40_^3−^ anions (*m/z*=607; *m*=mass and *z*=ionic charge) using a resolving quadrupole. The collisional quadrupole and resolving quadrupole are maintained at pressures of 2 × 10^−2^ and at 8 × 10^−5^ Torr, respectively. The mass-selected ions are then refocused with three einzel lenses in series before SL on the CNT electrode, which is mounted inside the vacuum deposition system. The collision energy of the soft-landed ions, determined by the difference between the CQ DC potential and the surface, was in a range of 30−35 eV per charge translating to an ion kinetic energy of ∼90 eV for PMo_12_O_40_^3−^. An ion current of ∼2 nA on the surface was measured with a picoammeter (model 9103, RBD Instruments, Bend, OR) and remained steady throughout the deposition. The total number of ions deposited was calculated using the measured ion current over time. The ion beam produced in the ion SL instrument is circular in shape and ∼3 mm in diameter. Therefore, the total deposition area is ∼7 mm^2^ on 100 mm^2^ CNT electrode.

*Ambient electrospray of PMo_12_O_40_*. Electrodes were also prepared by direct ambient ESD of 150 μM solutions of three different POMs (Na_3_[PMo_12_O_40_], (NH_4_)_3_[PMo_12_O_40_] and H_3_[PMo_12_O_40_]) with a fixed flow rate of 20 μl h^−1^ for a specific time period to deliver a predetermined amount of POM onto the CNT electrodes. POM solution was delivered to the ESD emitter through a fused silica capillary (inner diameter (ID): 100 μm, outer diameter (OD): 360 μm, Polymicro Technologies, Phoenix, AZ) using a syringe pump (KD Scientific, Holliston, MA). The emitter was produced by pulling a 500 μm OD four bore capillary (VitroCom, NJ, USA) to a final OD of 130 μm using a micropipette puller (*P*-2000, Sutter Instrument Company, Novato, CA). The ESD emitter was connected to the fused silica capillary using a microtight union (Upchurch Scientific). A voltage of ∼−2.5 kV was applied to the emitter to generate charged droplets. The electrode was positioned 3 mm away from the ESD emitter. It should be noted that ESD electrodes contain both anions ([PMo_12_O_40_]^*n*−^, *n*=2,3), counter cations (Na^+^, NH_4_^+^, H^+^) and solvent molecules while the SL electrodes contain only mass-selected [PMo_12_O_40_]^3−^ anions. See Fig. 1. The diameter of the deposition area is ∼3 mm. We found that the performance of the supercapacitor does not change with respect to the deposition spot size. However, we maintained the same deposition area for both SL and ESD during electrode preparation. The total number of ions deposited over time during ESD was calculated based on the flow rate and concentration of the POM solution.

### Preparation of electrolyte membrane

An ionic liquid-based electrolyte membrane was prepared using EMIMBF_4_ and copolymer poly(vinylidene fluoride-co-hexafluoropropylene) (PVDF-HFP). A typical preparation method is as follows: 2 g of PVDF-HFP was dissolved in 13 ml of DMF and stirred overnight at room temperature to make a homogenous solution. Two millilitre of EMIMBF_4_ ionic liquid was then added to the PVDF-HFP/DMF solution and stirred continuously for 4 h. Finally, 15 ml of EMIMBF_4_/PVDF-HFP solution was cast onto a 9 cm diameter petri dish and dried it in the oven at 70 °C for 12 h. The resulting membrane was peeled off and used as-prepared. See Supplementary Methods for detailed specifications of all chemicals used in this study.

### Fabrication and testing of supercapacitor devices

The supercapacitor devices were fabricated by placing an EMIMBF_4_/PVDF-HFP-based electrolyte membrane between two similar 1 cm × 1 cm as-prepared electrodes. The electrode assembly was gently pressed at 100 psi using a mechanical press (International Crystal Laboratories, Garfield, NJ) for 5 min and assembled in a two electrode testing cell specially designed for performing electrochemical measurements such as CV and GCD.

The GCD characteristics and stability of the supercapacitor devices were evaluated using a VersaSTAT 3 potentiostat/galvanostat (Princeton Applied Research, Oak Ridge, TN).The GCD experiments were carried out in the voltage range of 0–1 V at a current density of 8 A g^−1^—typical operating conditions used to study the performance of supercapacitors. Subsequently, the stability test was carried out by running the GCD experiment for 1,000 charge-discharge cycles.

Before performing GCD experiments, the intrinsic redox activity of PMo_12_O_40_ anions in EMIMBF_4_ was evaluated using CV to make sure that PMo_12_O_40_ anions are redox active in EMIMBF_4_ electrolyte. A CV was obtained on a GC working electrode containing 1 × 10^14^ Na_3_[PMo_12_O_40_] clusters deposited using ESD. Pristine EMIMBF_4_ was used as an electrolyte; platinum and silver wires were used as a counter and pseudo-reference electrodes, respectively.

The total specific capacitance (that is, the sum of capacitance contributions from non-faradaic reactions on carbon and faradaic reactions using the redox active material) and energy density of the supercapacitor were calculated using the following equations:


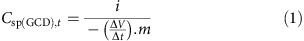



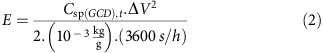



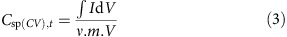



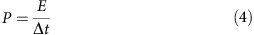


where, 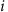
—current density used in the GCD experiment (8 A g^−1^); 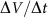
—slope of the discharge curve (V s^−1^); *C*_sp(GCD),*t*_ and *C*_sp(CV)*,t*_- total specific capacitance of the supercapacitor calculated from GCD and CV experiments respectively; Δ*V*—voltage difference between the voltage at the beginning of discharging and the voltage at the end of discharge in GCD measurement; *I* and *V*—response current (A) and potential window (V) in CV measurement, respectively; *v*—scan rate in the CV measurement; *m*—total mass of the active material on the electrode surface; *E*—energy density of the supercapacitor (Wh kg^−1^), *P*—Power density of the supercapacitor (kW kg^−1^), Δ*t*—discharge time (s).

To calculate the specific Faradaic capacitance of POM-based supercapacitors, it is assumed that only CNT present on the top layer contributes to the non-Faradaic double layer capacitance and the minor contribution from the layer behind the electrode interface is disregarded. Therefore, the combined weight of the CNT loading (∼25 μg cm^−2^) and the weight of immobilized [PMo_12_O_40_] were used as a total weight of the active material to calculate the total specific capacitance of the supercapacitor using equation 5.


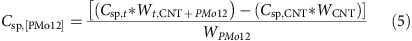


*C*_sp*,t*_—total specific capacitance (*F/g*) of modified CNT electrodes calculated using equation 1. *W*_*t*,CNT*+PMo1*2_—combined weight (*g*) of Faradaic and non-Faradaic active material (CNT loading ∼25 μg and loading of [PMo_12_O_40_] clusters in each case); *C*_sp,CNT_—total specific capacitance (*F/g*) of pristine CNT (only CNT as an active material); *W*_CNT_—weight (*g*) of CNT loading in a pristine CNT (∼25 μg); *W*_*PMo*12_
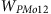
—mass of [PMo_12_O_40_] clusters at each loadings; *C*_sp,[PMo12]_—specific Faradaic capacitance contributed from [PMo_12_O_40_] clusters alone in the modified electrodes.

The oxidation state and surface characteristics of [PMo_12_O_40_] clusters on the different CNT electrodes were examined using XPS, SEM and STEM so that structural characteristics could be correlated with the electrochemical activity.

### Electrode characterization

*Scanning transmission electron microscopy*. The size and distribution of [PMo_12_O_40_] clusters on CNT electrodes were determined using STEM. In a typical sample preparation, a lift-out procedure was employed using a focused ion beam workstation, in which a portion of the CNT electrode was removed and transferred onto a copper TEM grid (Ted Pella, Inc., Redding, CA)^70^. STEM micrographs were then obtained using a FEI TITAN 80–300 eV TEM/STEM operated at 300 kV. The microscope is fitted with a spherical aberration corrector for the probe forming lens, enabling sub-Angstrom resolution in the STEM imaging mode. STEM analysis provides insight into the size and distribution of SL and ESD [PMo_12_O_40_] clusters on CNT electrodes through Z-contrast imaging in the HAADF mode. Approximately 1.5 × 10^13^ [PMo_12_O_40_] clusters were deposited using SL and ESD on CNT electrode and used for STEM analysis.

The STEM images were analysed as follows: the histograms of the cluster size distribution for SL and ESD samples were prepared with the Gatan Digital Micrograph software, which allows one to establish contrast threshold difference for Mo atoms. To analyse the STEM images of the SL sample (Fig. 3b), intact POM clusters and individual Mo atoms were identified and counted towards the generation of a histogram. A similar method was adopted for analysing images of ESD samples in Fig. 3c. These images were analysed by manually defining the features corresponding to both agglomerated POM clusters and individual Mo atoms and calculating the areas of these features. The average size of individual features shown in Fig. 3f was obtained assuming they have circular shapes.

*Scanning electron microscopy*. The surface morphology of different CNT electrodes was examined using a scanning electron microscope (Quanta 3D model, FEI, Inc.) operated at 10 kV accelerating voltage. Approximately 1.5 × 10^13^ [PMo_12_O_40_] clusters were deposited using SL and ESD on CNT electrodes for SEM analysis.

*X-ray photoelectron spectroscopy*. The oxidation states of Mo on different CNT electrodes were evaluated using Mo 3*d* XPS spectra. XPS measurements were performed with a Physical Electronics Quantera Scanning X-ray Microprobe. This system uses a focused monochromatic Al Kα X-ray (1,486.7 eV) source for excitation and a spherical section analyser. The instrument has a 32 element multichannel detection system. A 100 W X-ray beam focused to 100 μm diameter was rastered over a 1.2 mm × 0.1 mm rectangular region on the sample. The X-ray beam is incident normal to the sample and the photoelectron detector is at 45° off-normal. High energy resolution spectra were collected using a pass-energy of 69.0 eV with a step size of 0.125 eV. For the Ag 3*d*_5/2_ line, these conditions produced a FWHM of 0.91 eV. Approximately 1.5 × 10^13^ [PMo_12_O_40_] clusters were deposited using SL and ESD on CNT electrode and used for XPS analysis. The XPS spectra were fitted using Multipak Spectrum software and the following peak fitting parameters were used. The intensity ratio of Mo 3*d*_5/2_ and Mo 3*d*_3/2_ peaks arising from spin–orbit coupling was fixed at 2:3 corresponding to d orbital and the spit–orbit splitting was fixed at 3.1 eV. The Shirley background subtraction method with 80% Gaussian, 20% Lorentz line shapes was employed to fit both Mo 3*d* and C 1s XPS spectra.

## Additional information

**How to cite this article:** Prabhakaran, V. *et al*. Rational design of efficient electrode-electrolyte interfaces for solid-state energy storage using ion soft-landing. *Nat. Commun.* 7:11399 doi: 10.1038/ncomms11399 (2016).

## Supplementary Material

Supplementary Information Supplementary Figures 1-16, Supplementary Discussion, Supplementary Methods and Supplementary References

## Figures and Tables

**Table 1:** XPS analysis results of Mo 3*d* spectra obtained on the CNT-carbon electrodes modified with POM anions.

**Binding energy (eV)**	**Oxidation state**		**Area %**			
			**SL-CNT**	**ESD-NaPOM-CNT**	**ESD-NH**_**4**_**POM-CNT**	**ESD-HPOM-CNT**
232.0	Mo 3*d*_5/2_	5+	8.1	6.7	3.0	6.8
233.1		6+	52.0	53.4	57.0	53.4
235.1	Mo 3*d*_3/2_	5+	5.4	4.4	2.0	4.5
236.2		6+	34.6	35.6	38.0	35.5
	**FWHM (eV)**		1.6 eV	1.4 eV	1.3 eV	1.4 eV

CNT, carbon nanotube; ESD, electrospray deposition; FWHM, full-width at half-maximum; POM, polyoxometalate; SL, soft landing; XPS, X-ray photoelectron spectroscopy.
