# Supplementary material for: Rational design of efficient electrode–electrolyte interfaces for solid-state energy storage using ion soft landing
Source: Nat Commun. 2016 Apr 21;7:11399. doi: 10.1038/ncomms11399 (PMC4844687; doi:10.1038/ncomms11399)
Supplement: Supplementary Information — Supplementary Figures 1-16, Supplementary Discussion, Supplementary Methods and Supplementary References [file ncomms11399-s1.pdf]

Supplementary Figures

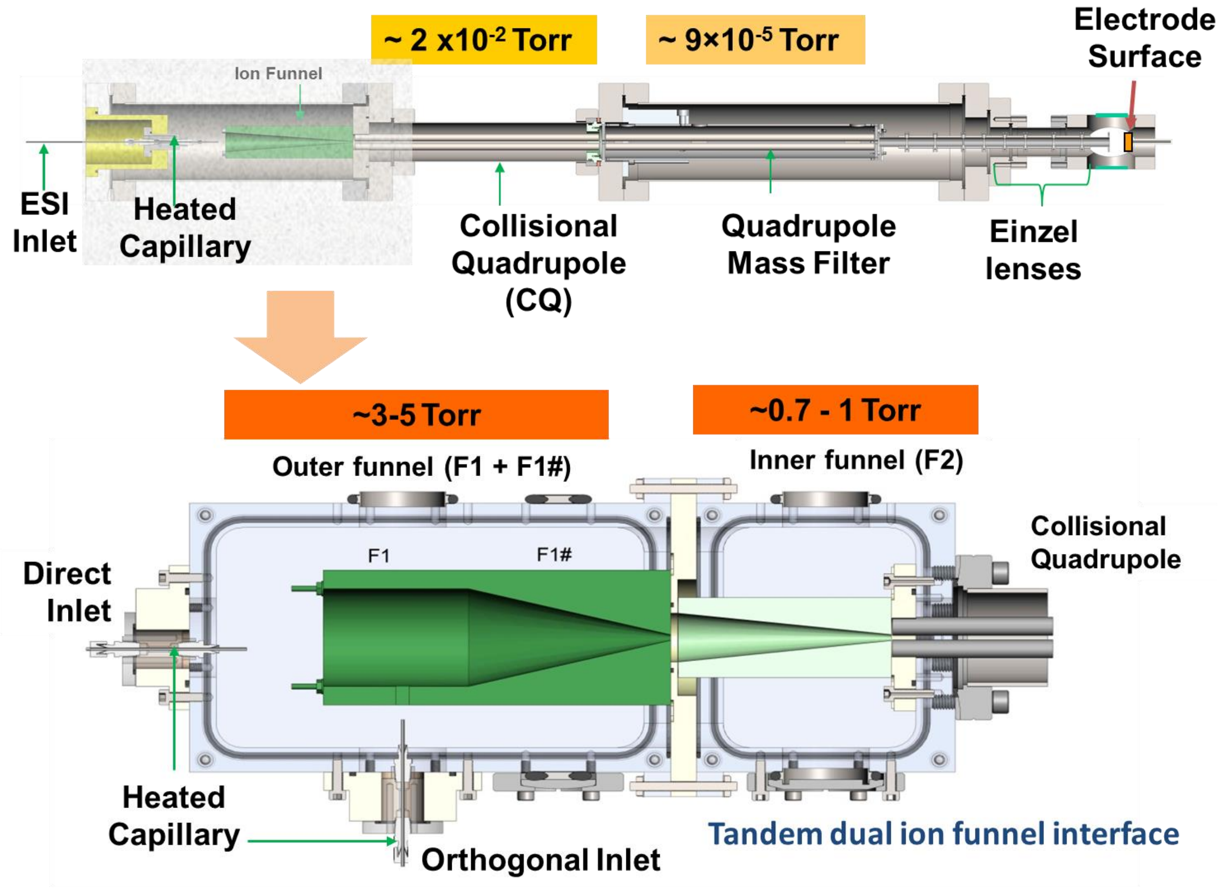

Supplementary Figure 1. Ion soft-landing instrument. Schematic diagram of the high flux ion soft-landing instrument employed in this study.[1]

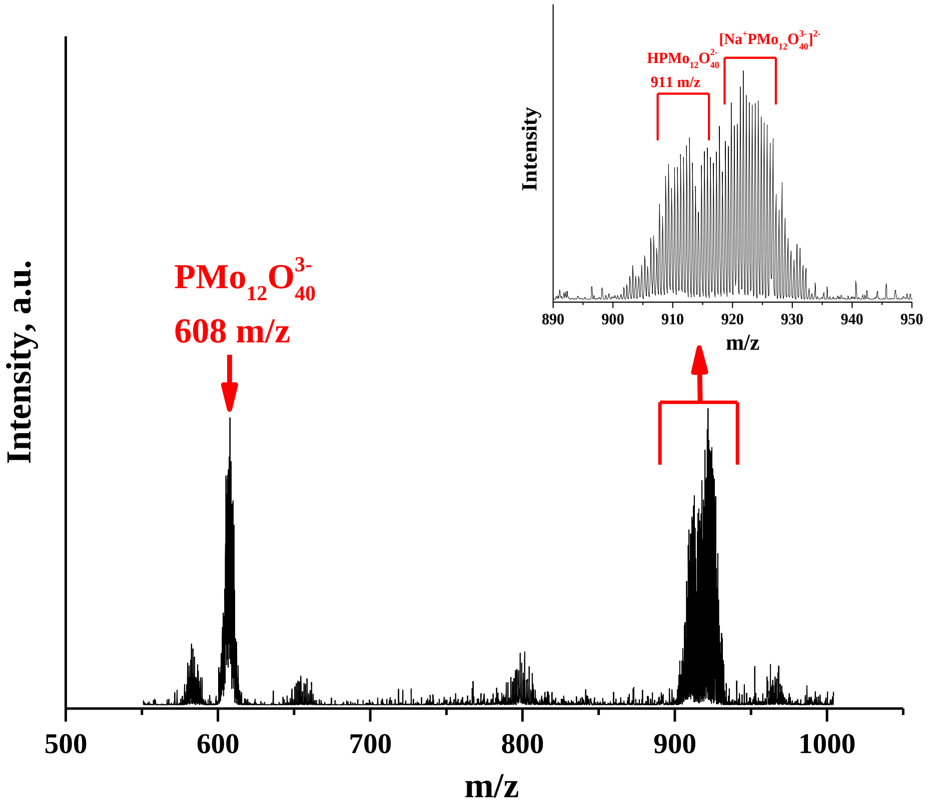

Supplementary Figure 2. Mass spectrum of electrosprayed [$PMo_{12}O_{40}$] solution. Representative mass spectrum showing molydenum polyoxometalate [$PMo_{12}O_{40}$] anions - [$PMo_{12}O_{40}$]$^{3-}$, [$HPMo_{12}O_{40}$]$^{2-}$, and [$NaPMo_{12}O_{40}$]$^{2-}$ are the major anions produced by electrospray ionization (m = mass and z = charge). Insert shows neighboring doubly charged [$PMo_{12}O_{40}$] anions with and without a sodium cation.

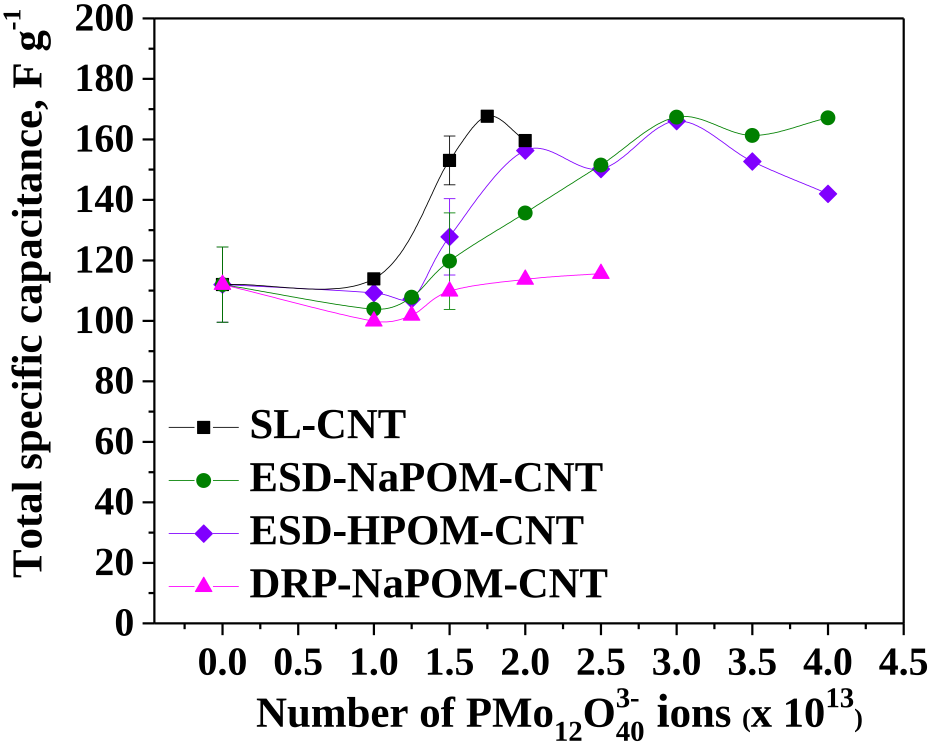

Supplementary Figure 3: Electrochemical performance of supercapacitors fabricated with different methods. Effect of POM loading on the total specific capacitance of supercapacitors fabricated using ion soft landing (SL), ambient electrospray deposition (ESD), and ambient drop casting (DRP).

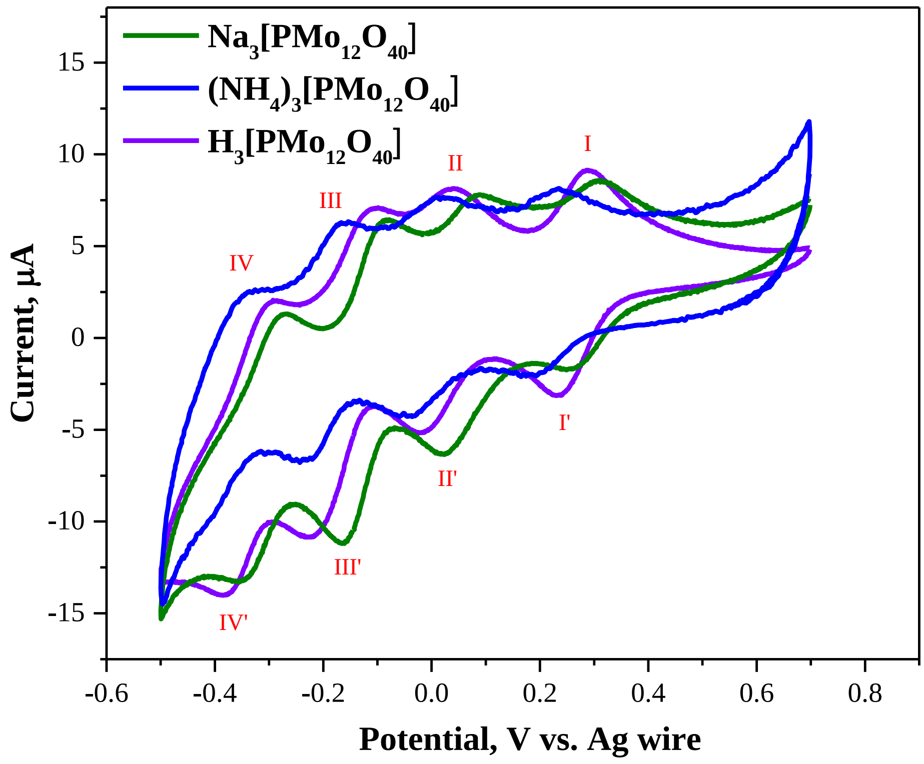

Supplementary Figure 4. Redox properties of different $[PMo_{12}O_{40}]$ salts. Cyclic voltammograms (CVs) of $Na_3[PMo_{12}O_{40}]$, $(NH_4)_3[PMo_{12}O_{40}]$, and $H_3[PMo_{12}O_{40}]$. Working electrode: polished glassy carbon; counter electrode: Pt wire; pseudo-reference electrode: Ag wire. Scan rate: 50 mV s^{-1}. CVs were measured using 500 µM solution of $Na_3[PMo_{12}O_{40}]$, $(NH_4)_3[PMo_{12}O_{40}]$ and $H_3[PMo_{12}O_{40}]$ salts in $EMIMBF_4$ IL electrolyte. The CV was performed at an applied potential between -0.5 V and +0.7 V vs. Ag wire at a scan rate of 50 $mVs^{-1.}$ The CV reveals four characteristic redox peaks denoted by I/I', II/II', III/III' and IV/IV', respectively (refer Supplementary Fig. 3). The electron number ($\Delta(E_{p,ox} - E_{p,red})$ is found to be ~1 in each case, corresponds to a reversible one electron transfer process. The potential shift of ~30 mV and ~70 mV in the redox peaks of $H_3[PMo_{12}O_{40}]$ and $(NH_4)_3[PMo_{12}O_{40}]$ respectively observed with respect to $Na_3[PMo_{12}O_{40}]$ was attributed to the effect of the presence of different counter cations in each case.[2] These observations confirm the existence of the characteristic redox activity of $[PMo_{12}O_{40}]$ in the $EMIMBF_4$ ionic liquid.

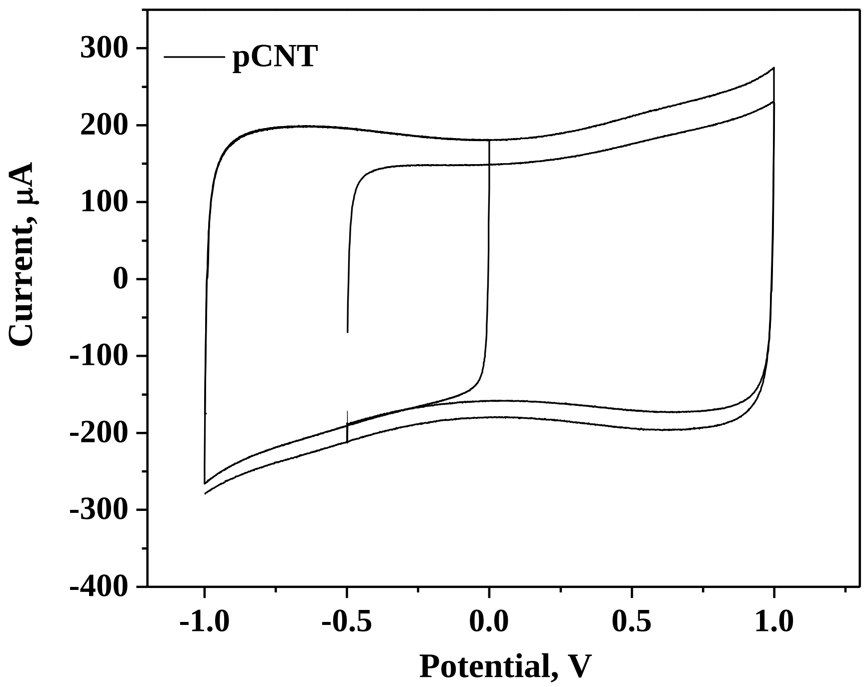

Supplementary Figure 5. Potential stability of fabricated supercapacitor with pCNT. Representative CVs of a supercapacitor assembled with pCNT performed between different potential ranges, -1.0 V to 0 V, -0.5 V to 1.0 V and -1.0 V to 1.0 V to demonstrate the stability of the electrolyte and the fabricated supercapacitor. Scan rate: 50 mV s^{-1}. An $EMIMBF_4$ based membrane was used as a separator. A rectangular shape of the CV confirms the stability of the electrolyte and the fabricated supercapacitor over wide potential ranges.

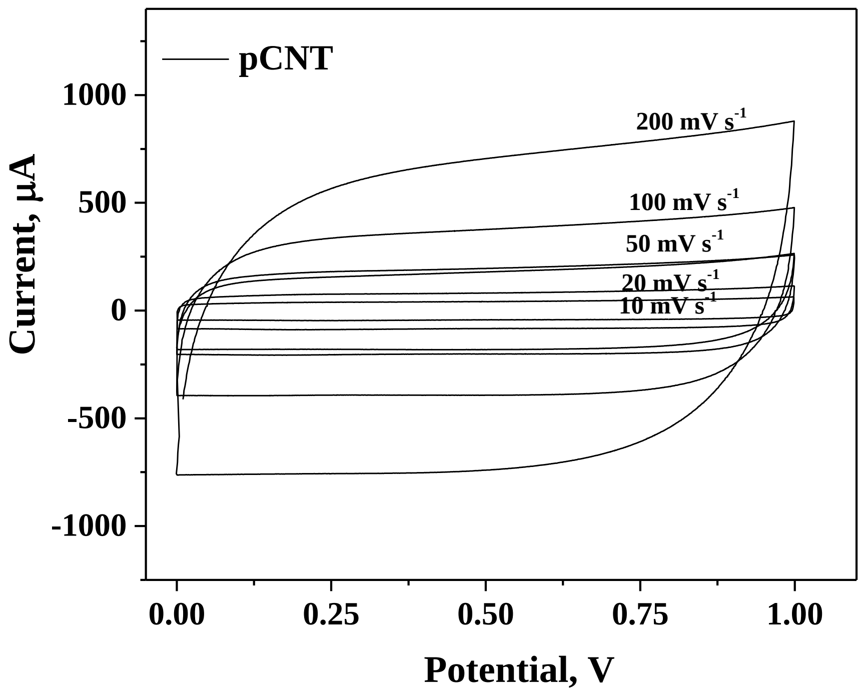

Supplementary Figure 6. CVs of pCNT at different scan rates. Representative CVs of a supercapacitor assembled with pCNT electrodes performed at different scan rates, 10, 20, 50, 100 and 200 mV s^{-1}. An $EMIMBF_4$ based membrane was used as a separator.

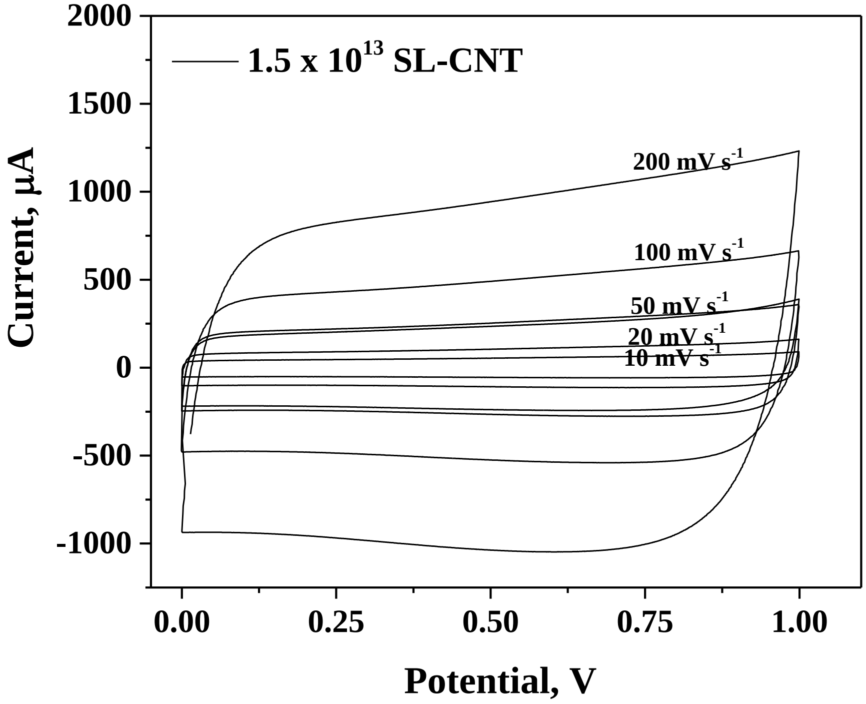

Supplementary Figure 7. CVs of SL-CNT at different scan rates. Representative CVs of a supercapacitor assembled with electrodes modified with SL-CNT performed at different scan rates, 10, 20, 50, 100 and 200 mV s^{-1}, An $EMIMBF_4$ based membrane was used as a separator.

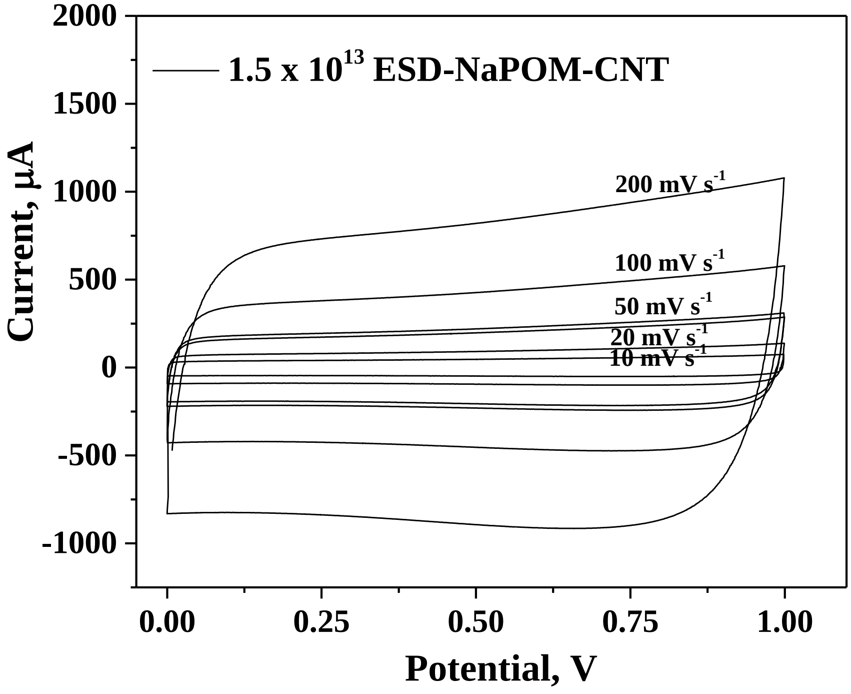

Supplementary Figure 8. CVs of ESD-NaPOM-CNT at different scan rates. Representative CVs of a supercapacitor assembled with ESD-NaPOM-CNT performed at different scan rates, 10, 20, 50, 100 and 200 mV s^{-1}. An $EMIMBF_4$ based membrane was used as a separator.

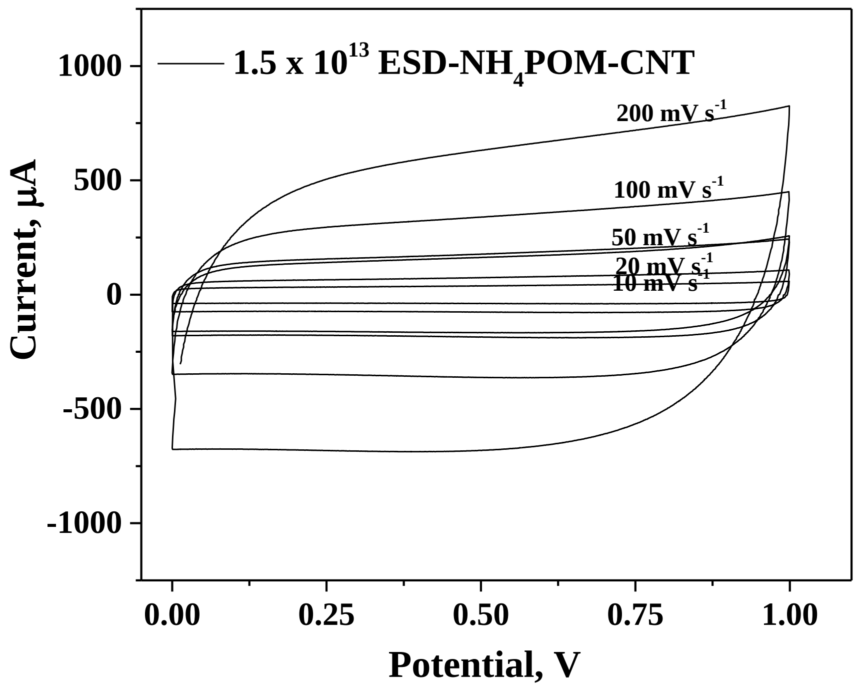

Supplementary Figure 9. CVs of ESD-NH_4POM-CNT at different scan rates. Representative CVs of a supercapacitor assembled with ESD-NH_4-CNT performed at different scan rates, 10, 20, 50, 100 and 200 mV/s. An $EMIMBF_4$ based membrane was used as a separator.

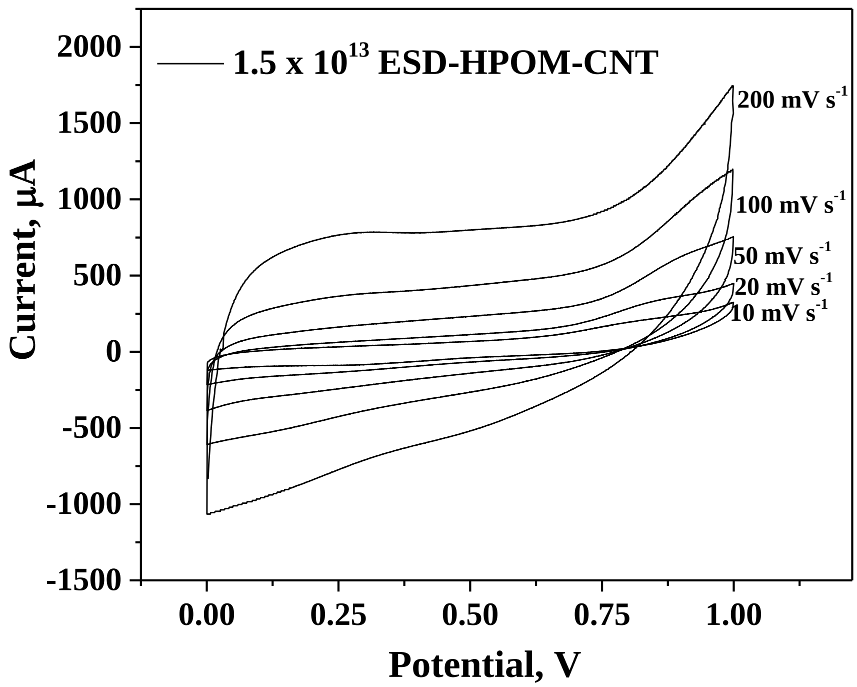

Supplementary Figure 10. CVs of ESD-HPOM-CNT at different scan rates. Representative cyclic voltammograms of a supercapacitor assembled with ESD-HPOM-CNT performed at different scan rates, 10, 20, 50, 100 and 200 mV s^{-1}. An $EMIMBF_4$ based membrane was used as a separator.

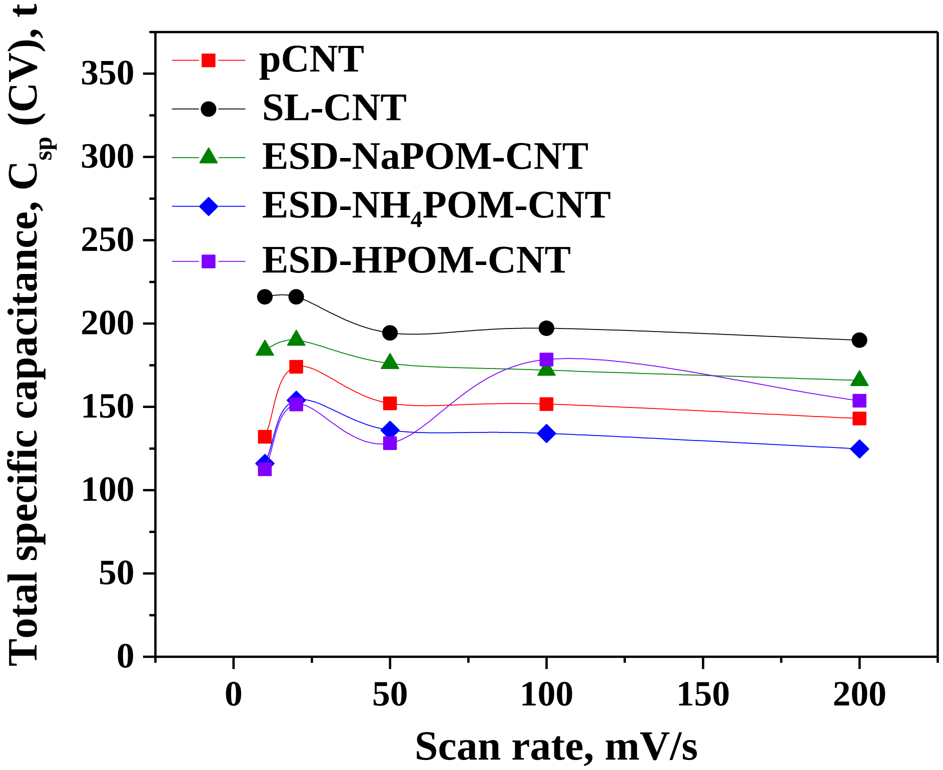

Supplementary Figure 11. Relation between specific capacitance and scan rates. Comparison of the total specific capacitance of supercapacitors calculated using the data obtained from CVs at different scan rates (10, 20, 50, 100 and 200 mV s^{-1}).

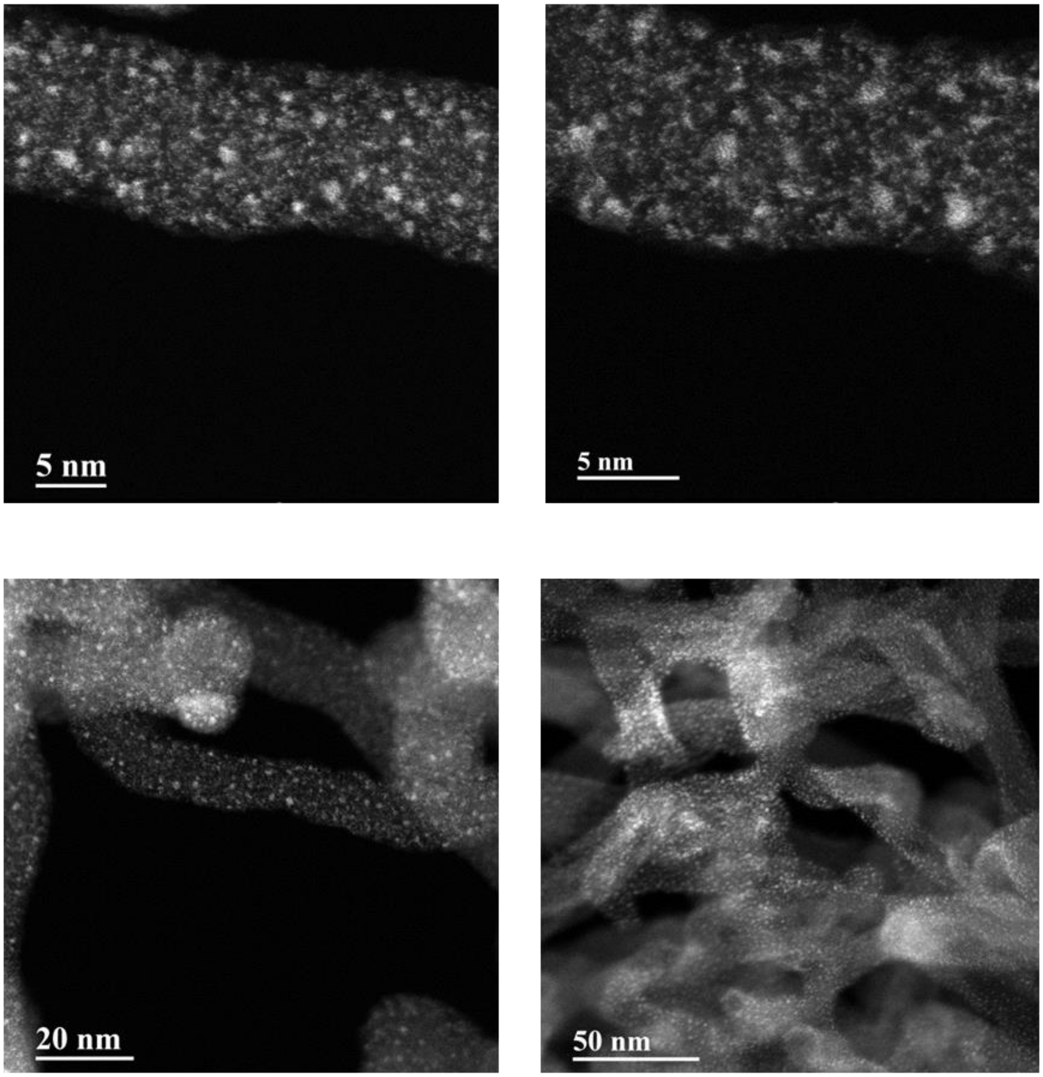

Supplementary Figure 12. HAADF-STEM images of POM on SL-CNT. HAADF-STEM images of SL-CNT showing homogenous distribution of POM on CNTs.

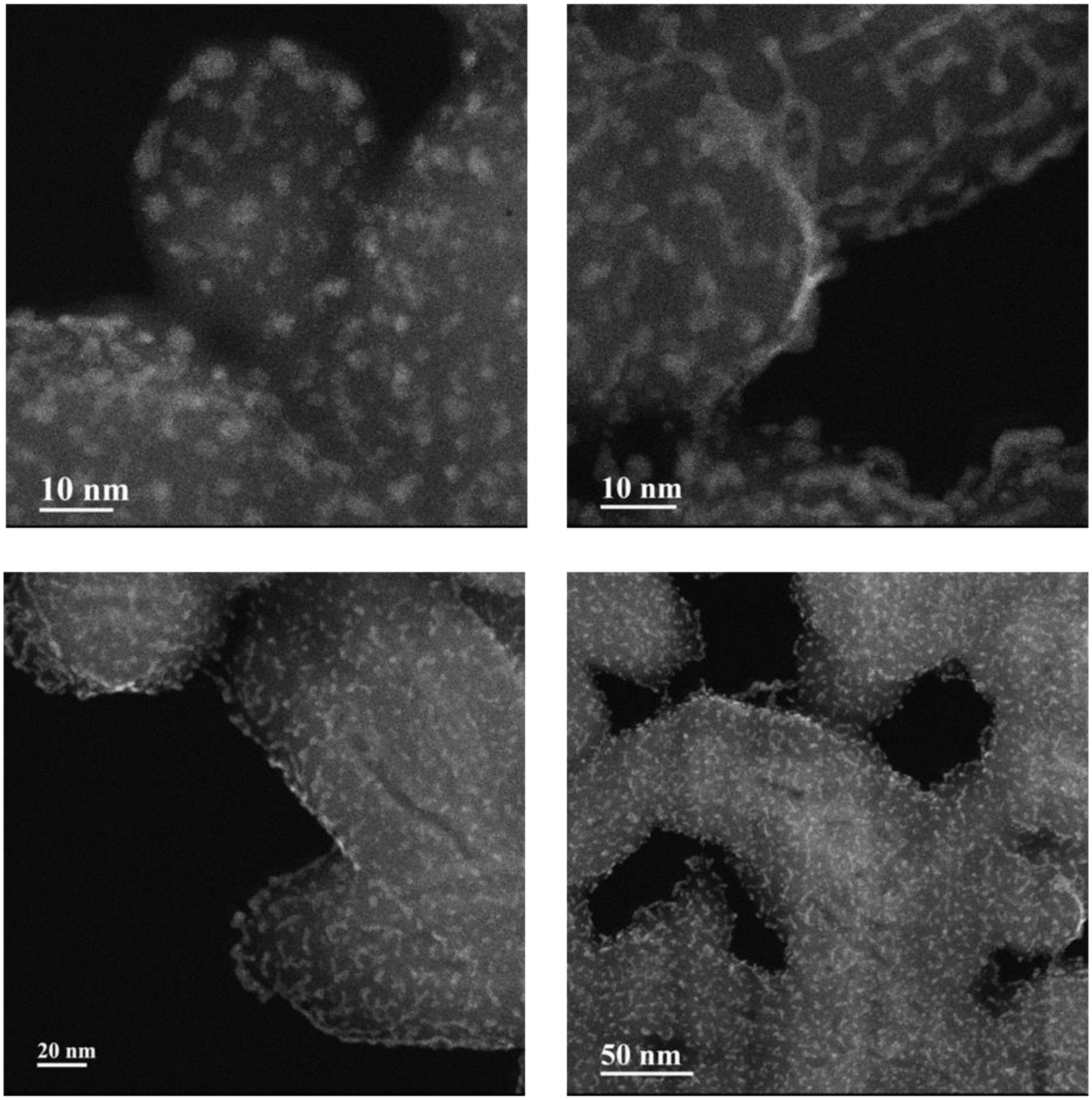

Supplementary Figure 13. HAADF-STEM images of POM on ESD-NaPOM-CNT. HAADF-STEM images of ESD-NaPOM-CNT showing aggregates of POM at different sample locations.

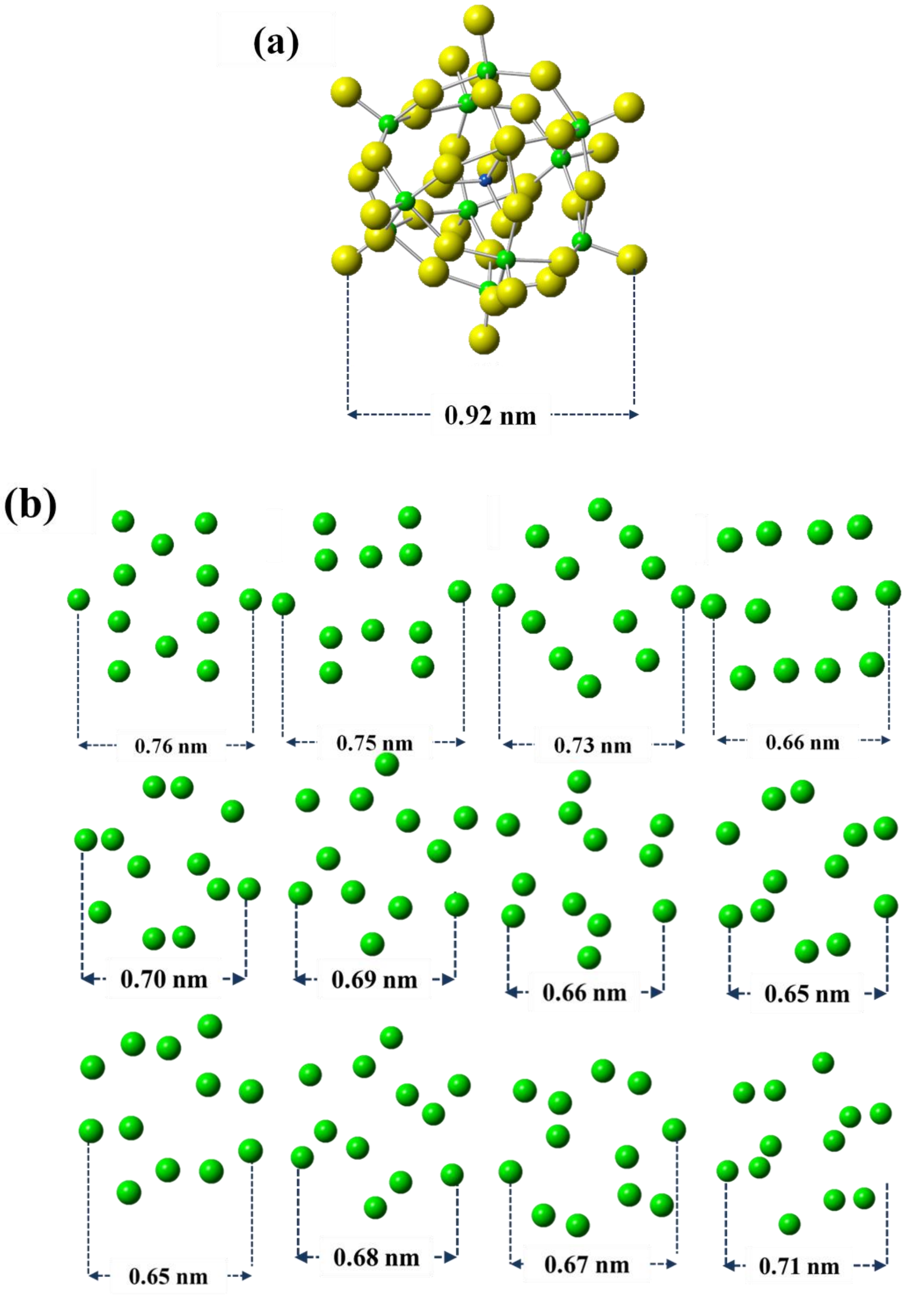

Supplementary Figure 14. Structures of Keggin [$PMo_{12}O_{40}$] clusters with different orientations. (a) Molecular structure of Keggin [$PMo_{12}O_{40}$] cluster, (b) Examples of Keggin cluster with different orientations and diameters (only Mo atoms are visible). **Green**= Molydenum, Yellow=Oxygen, Blue=Phosphorous. The first row represents the [$PMo_{12}O_{40}$] cluster with symmetrical orientation and the second and the third rows represent the [$PMo_{12}O_{40}$] cluster with random orientation. SL [$PMo_{12}O_{40}$] clusters deposited on CNT surface have a combination of symmetrical and randomly oriented clusters.

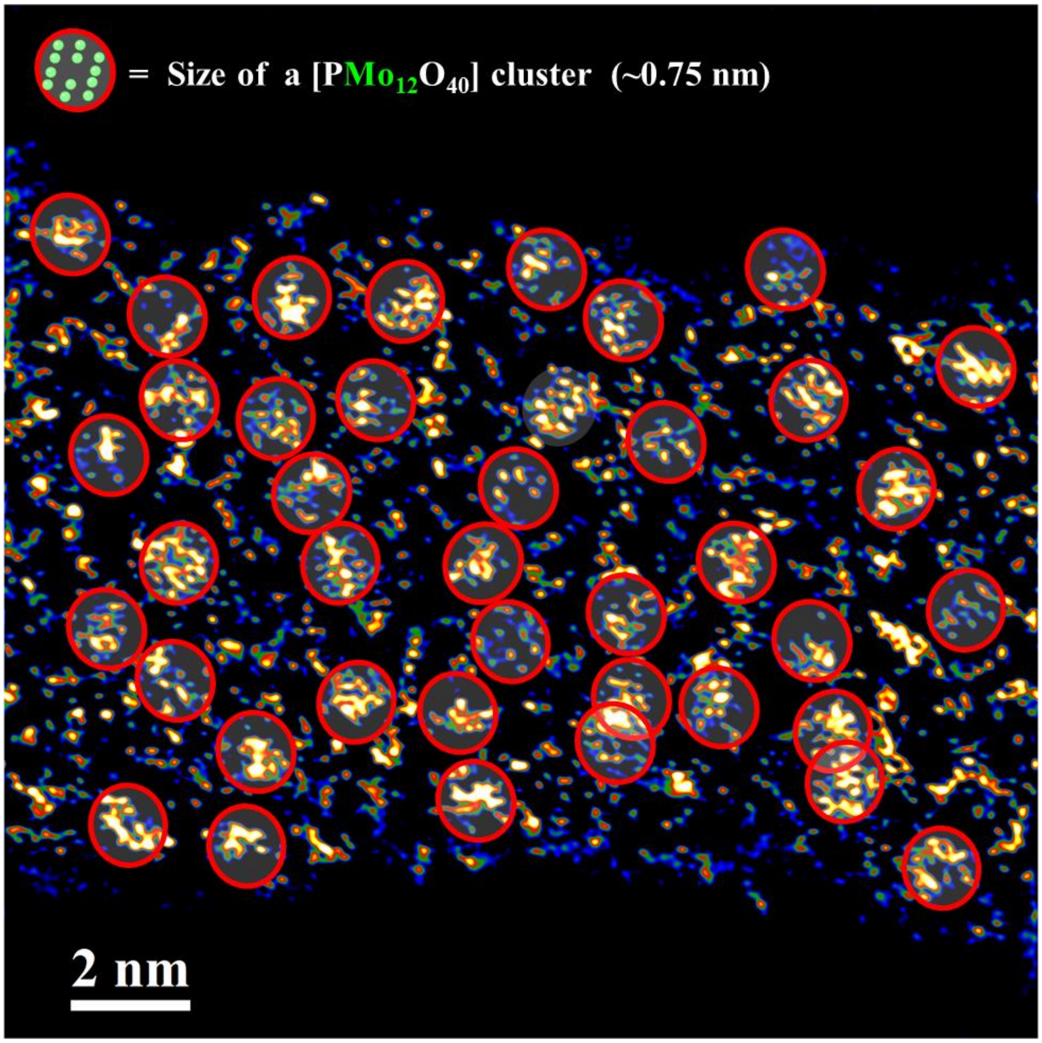

Supplementary Figure 15. Identification of intact [$PMo_{12}O_{40}$] clusters. Map of Keggin [$PMo_{12}O_{40}$] clusters in the HAADF STEM image of SL-CNT. The intact clusters were marked with red circles.

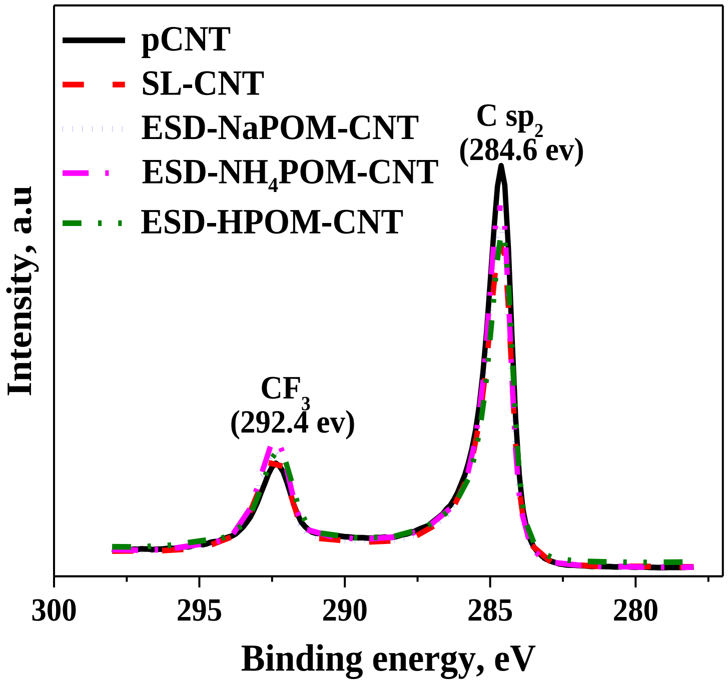

Supplementary Figure 16. C 1s spectra of different CNT electrodes. C 1s spectra of pCNT, SL-CNT, ESD-NaPOM-CNT, ESD-NH_4POM-CNT and ESD-HPOM-CNT. C1s spectra of all electrodes have a characteristic peak at 284.6 eV corresponding to linear C sp2 present in the CNT-carbon electrodes. No peak shift was observed between unmodified and modified electrodes indicating that there is no charge transfer interaction between CNT-carbon and $[PMo_{12}O_{40}]$,[3] or at least, it is not observed due to low coverage of $[PMo_{12}O_{40}]$ on CNT. Another peak at 292.4 eV corresponds to the presence of CF_3. CF_3 is carried over from polytetrafluoroethylene (PTFE) present in the CNT-carbon electrode surface. PTFE coating was applied on the electrodes during the manufacturing process to make the surface more hydrophobic. PTFE is, however, an electrochemically inactive neutral species and expected to remain inactive during electrochemical processes.

Supplementary Discussion

Calculation of coverage of [$PMo_{12}O_{40}$] from STEM analysis

To calculate the coverage from the STEM image, the total number of individual [$PMo_{12}O_{40}$] clusters was identified by mapping individual Keggin [$PMo_{12}O_{40}$] clusters with different orientations onto the STEM image (Refer Supplementary Fig.14 for a few examples of Keggin structures with different orientations and cluster sizes and Supplementary Fig. 15 for a mapped STEM image). During the mapping process, it was also observed that some of the Mo atoms did not appear to exist in the Keggin structure, rather they exist as separated individual atoms. This finding may be attributed to several factors: (i) fragmentation of a small fraction of [$PMo_{12}O_{40}$] clusters during ion soft landing (ii) 0.75 nm sized Keggin [$PMo_{12}O_{40}$] clusters were deposited on three dimensional 10 nm diameter CNT support, therefore, some clusters may sit on the side walls of CNT. In this case, only a fraction of a cluster with this shielded orientation may be exposed to STEM imaging. It is very difficult to predict the conclusive cause of existence of individual Mo atoms. For calculation purposes, every 12 individual Mo atoms were considered as one [$PMo_{12}O_{40}$] cluster.

The STEM image of SL [$PMo_{12}O_{40}$] cluster presented in Fig. 3 (b) is examined for this calculation. The analysis revealed that there are 39 individual [$PMo_{12}O_{40}$] clusters and 179 Mo atoms. Assuming that 179 Mo atoms are also a portion of [$PMo_{12}O_{40}$] clusters and 12 Mo atoms are present in a [$PMo_{12}O_{40}$] cluster, the total number of [$PMo_{12}O_{40}$] clusters is 55 (See Supplementary Fig.15)

CNT support in Fig. 3 (a) has 10 nm diameter and 17 nm length. Therefore, the total surface area of semi-CNT wall is 267 nm^2.

Thus,

$$\text{Coverage of } [\mathrm{PMo}_{12}O_{40}]\,clusters\ on\ CNT = \frac{Total\ number\ of\ [\mathrm{PMo}_{12}\mathrm{O}_{40}]\mathrm{clusters}}{Total\ area\ of\ the\ CNT\ support}$$

$$= \frac{55\ clusters}{267\ nm^2}$$

$= 0.21\frac{cluster}{nm^2}$ = 2.1 x 10^5 [$PMo_{12}O_{40}$] clusters μm^{-2}.

Supplementary Methods

Chemicals

1-Ethyl-3-methylimidazolium tetra fluoroborate ($EMIMBF_4$) (99%), poly(vinylidene fluoride-co-hexafluoropropylene) (PVDF-HFP) (ACS grade), sodium phosphomolybdate hydrate ($Na_3[PMo_{12}O_{40}]$ x H_2O) (98%), ammonium phosphomolybdate hydrate ($(NH_4)_3[PMo_{12}O_{40}]$ x H_2O) (61%), and anhydrous N,N-dimethylformamide (99.8 %) were all purchased from Sigma-Aldrich (St. Louis, MO) and used without further purification.

Supplementary References

1 Gunaratne, K. D. D. *et al.* Design and performance of a high-flux electrospray ionization source for ion soft landing. *Analyst* **140**, 2957-2963 (2015).

2 Song, I. K., Kim, H. S. & Chun, M.-S. On the reduction potential of cation-exchanged heteropolyacids (HPAs). *Korean Journal of Chemical Engineering* **20**, 844-849.

3 Bosch-Navarro, C. *et al.* Charge transfer interactions in self-assembled single walled carbon nanotubes/Dawson–Wells polyoxometalate hybrids. *Chemical Science* (2014).
